# Cholinergic Modulation of Cortical Microcircuits Is Layer-Specific: Evidence from Rodent, Monkey and Human Brain

**DOI:** 10.3389/fncir.2017.00100

**Published:** 2017-12-08

**Authors:** Joshua Obermayer, Matthijs B. Verhoog, Antonio Luchicchi, Huibert D. Mansvelder

**Affiliations:** Department of Integrative Neurophysiology, Center for Neurogenomics and Cognitive Research, Neuroscience Amsterdam, VU University Amsterdam, Amsterdam, Netherlands

**Keywords:** acetylcholine, neocortex, microcircuits, pyramidal neuron, interneurons, synaptic transmission, synaptic plasticity, basal forebrain

## Abstract

Acetylcholine (ACh) signaling shapes neuronal circuit development and underlies specific aspects of cognitive functions and behaviors, including attention, learning, memory and motivation. During behavior, activation of muscarinic and nicotinic acetylcholine receptors (mAChRs and nAChRs) by ACh alters the activation state of neurons, and neuronal circuits most likely process information differently with elevated levels of ACh. In several brain regions, ACh has been shown to alter synaptic strength as well. By changing the rules for synaptic plasticity, ACh can have prolonged effects on and rearrange connectivity between neurons that outlasts its presence. From recent discoveries in the mouse, rat, monkey and human brain, a picture emerges in which the basal forebrain (BF) cholinergic system targets the neocortex with much more spatial and temporal detail than previously considered. Fast cholinergic synapses acting on a millisecond time scale are abundant in the mammalian cerebral cortex, and provide BF cholinergic neurons with the possibility to rapidly alter information flow in cortical microcircuits. Finally, recent studies have outlined novel mechanisms of how cholinergic projections from the BF affect synaptic strength in several brain areas of the rodent brain, with behavioral consequences. This review highlights these exciting developments and discusses how these findings translate to human brain circuitries.

## Introduction

Neuromodulation of the neocortex by acetylcholine (ACh) not only shapes neuronal circuit development, but is also crucial for sensory and cognitive behavior, such as sensory detection, attention, learning and memory (Dalley et al., 2004; Hasselmo and Giocomo, 2006; Sarter et al., 2009). Due to the strong impact of cholinergic modulation of the neocortex and its role in neuropsychiatric disorders, it is imperative that we understand the neuronal and synaptic mechanisms underlying ACh’s role in cognition and neocortical microcircuit function. Despite the fact that we know for instance that cholinergic signaling in the prefrontal cortex (PFC) is involved in attention, very little is known about the neuronal circuit mechanisms involved. How do cholinergic receptors expressed by specific types of neurons contribute to sensory processing, attention or working memory? Many studies emphasize the sustained ACh effects, in which ACh acts as a slow neuromodulator increasing excitability of networks (Picciotto et al., 2012). However, ACh does not only have tonic neuromodulatory roles changing cortical states, but also mediates specific cognitive operations (Howe et al., 2013). At the microcircuit level, it is becoming clear that ACh is not only a slow neuromodulator, but recent evidence shows that it can instantly alter information flow by direct, fast point-to-point ACh synapses that target specific pyramidal neurons and interneurons and act on millisecond time scales (Arroyo et al., 2014). Moreover, recent exciting discoveries show that the basal forebrain (BF) cholinergic system may be innervating the neocortex at a spatially more refined scale than previously considered, raising the possibility that a more fine-scaled control by ACh exists that does not only confer specificity on a brain area scale, but even on that of cortical lamina. Finally, recent findings on cholinergic modulation of human neocortical microcircuits suggest that also in the human brain these mechanisms exist, which may prompt us to change our view on the cholinergic system as being merely a slow acting arousal system to one that includes a fast acting manipulation of cortical information flow important for sub-second cognitive operations. This review highlights recent evidence from rodent, monkey and human brain that show that the cholinergic system in the mammalian brain acts on a spatial and temporal scale that is much more detailed than previously considered.

## Specificity of Basal Forebrain Cholinergic Projections to the Neocortex

All areas of the human cerebral cortex contain cholinergic axons, but higher densities of cholinergic axons exist in limbic and paralimbic cortical areas, such as hippocampus and amygdala, than in primary sensory-motor and sensory association cortex (Mesulam et al., 1992a,b; Smiley et al., 1997). Regional variation in cholinergic innervation of the human cerebral cortex seems to follow the organization of information processing systems, with lower innervation in primary sensory areas and increasing innervation in higher order processing areas (Mesulam et al., 1992a). Both in the primate and rodent brain, the main cholinergic innervation of the neocortex originates in the BF (Woolf and Butcher, 2011; Mesulam, 2013). In addition, there are sparse local cholinergic interneurons throughout the cortex, but it is still undetermined whether ACh release from these neurons actually occurs (von Engelhardt et al., 2007). Several BF nuclei containing cholinergic neurons project to cortical and sub-cortical target areas. In the human and non-human primate brain, four BF regions with cholinergic neurons have been identified (Mesulam et al., 1983; Zaborszky et al., 2008; Mesulam, 2013), of which the nucleus basalis (NB) cholinergic neurons (substantia innominata (SI), Ch4) innervate the neocortex (Mesulam and Geula, 1988). Medial septal nucleus cholinergic neurons (Ch1), the vertical limb of the diagonal band (Ch2) and the horizontal limb of the diagonal band (Ch3) project to the hippocampus, hypothalamus, olfactory bulb and other brain areas (Mesulam, 2013). Both in primates as well as in rodents, neocortical innervation by the BF cholinergic system is topographically organized, suggesting that functional control of neocortical processing by the BF cholinergic system can be very specific. In the macaque brain, cholinergic neurons located in different parts of the NB project to distinct cortical areas. For example, the anteromedial subdivision of Ch4 is the major cholinergic input source to medial cortical areas, such as the cingulate gyrus; the anterolateral Ch4 subdivision to frontoparietal cortex, opercular regions, and the amygdaloid nuclei; intermediate Ch4 subdivision to laterodorsal frontoparietal, peristriate, and midtemporal regions; and posterior subdivision of Ch4 to the superior temporal and temporopolar areas (Mesulam et al., 1983).

In the rodent brain, a detailed topographical organization of the BF cholinergic neurons exists (Bigl et al., 1982; Lamour et al., 1982; Price and Stern, 1983; Gritti et al., 2003; Bloem et al., 2014; Zaborszky et al., 2015; Kondo and Zaborszky, 2016). Early studies indicated that in rat brain, large but discrete cortical areas are innervated by small groups of cholinergic BF neurons. Cholinergic neurons in the diagonal band of Broca tend to innervate the cingulate and occipital cortices. The SI cholinergic neurons project more to the frontal cortex, while the cholinergic cells in the globus pallidus seem to target the temporal and parietal cortices (Lamour et al., 1982; Price and Stern, 1983; Rye et al., 1984). More recent studies show that also on a finer scale, within brain regions such as the PFC and the parahippocampal cortex, a topographical mapping between BF cholinergic neurons and neocortical areas exists (Bloem et al., 2014; Zaborszky et al., 2015; Kondo and Zaborszky, 2016). Cholinergic neurons that innervate the PFC show a frontal-caudal gradient in the location of the cell bodies of these neurons in the BF. Using an anterograde viral labeling approach in mouse brain, it was found that cholinergic neurons located at rostral locations in the BF, in particular in the horizontal limb of the diagonal band (HDB), innervate predominantly rostral and ventral medial prefrontal cortical (mPFC) areas, whereas caudo-lateral neurons in the BF, such as the SI and NB, preferentially innervate the dorsal and caudal mPFC regions (Bloem et al., 2014). These distinct BF regions send projections to the neocortex through distinct pathways (Bloem et al., 2014), as was also shown in rat (Saper, 1984; Luiten et al., 1987; Eckenstein et al., 1988). In rat brain, using injection of retrograde tracers in anterior cingulate cortex (ACC), mPFC and orbitofrontal cortex, the topographic organization of BF cholinergic projections was less pronounced. Approximately 60% of neurons in the BF targets more than one of these areas and twenty percent of neurons innervates all three areas (Chandler and Waterhouse, 2012; Chandler et al., 2013; Zaborszky et al., 2015). The apparent discrepancy in topographic organization of mouse and rat BF likely results from retrograde vs. anterograde tracing methods. Retrograde tracing methods can show that neurons project to a certain brain region, but they fail to determine projection density. In contrast, projection density can be determined using an anterograde approach (Bloem et al., 2014). Injecting an anterograde tracer in the magnocellular preoptic nucleus (MCPO) and SI in rat (Henny and Jones, 2008) it was shown that labeling was strongest in the infralimbic (IL) PFC. This suggests that the topographic mapping from BF cholinergic neurons to PFC is revealed by taking innervation densities into account (Wouterlood et al., 2014). Thus, although BF neurons in the rodent brain often project to multiple regions of the PFC, they preferentially innervate different regions based on their location in the BF (Bloem et al., 2014).

Traditionally, the anatomy of the BF cholinergic system has been studied with retrograde labeling, for instance horse radish peroxidase (HRP) injections in neocortical target areas or with antibody staining for cholineacetyl transferase (ChAT) or acetylcholine esterase (AChE; Lamour et al., 1982; Rye et al., 1984; Mesulam, 2013). With these approaches, it is virtually impossible to establish whether individual BF cholinergic neurons target specific cortical layers. Using an anterograde viral labeling strategy based on cre-recombinase-dependent expression of GFP/YFP in ChAT-cre transgenic mice it is possible to address this issue (Wouterlood et al., 2014), and we recently found that different locations in the BF specifically innervate superficial or deep lamina of PFC (Bloem et al., 2014). In superficial layers 1–3, a marked distinction between different injection sites was found, particularly in prelimbic (PL), IL and the ventral part of the anterior cingulate PFC. Cholinergic neurons in the rostral part of the BF project fibers to both superficial layers and deep layers of the mPFC. In stark contrast, cholinergic neurons in caudal parts of the BF preferentially projected to deep layers of the mPFC and hardly innervate the superficial layers (Bloem et al., 2014). This suggests that two separate populations of BF neurons send cholinergic projections to the PL, IL and ACv, one that innervates all layers and another that selectively targets deep layers.

In mouse brain, cholinergic fibers travel via four routes from the BF (medial, septal, internal capsule and lateral) to cortical targets (Bloem et al., 2014). Cholinergic fibers enter the neocortex either via layer 1 or layer 6. Interestingly, fibers arising from rostral parts show a large number of fibers taking the medial route and stronger innervation of mPFC superficial layers. Caudal cholinergic neurons do not send projections through the medial route, and enter the neocortex through layer 6, predominantly targeting deep layers. These specific projection profiles of cholinergic neurons at different locations of the BF may highlight functional differences between rostral and caudal BF regions and the pathways innervating cortex through L1 and L6.

Similar to the BF to PFC cholinergic system, a topographical organization also occurs between the BF and the parahippocampal cortex (Kondo and Zaborszky, 2016). The BF sends complementary projections to perirhinal, postrhinal, and entorhinal cortex. The perirhinal and postrhinal cortex receive cholinergic projections predominantly from caudal BF regions: the caudal globus pallidus and SI and HDB. In contrast, the rostral part of the BF, including the medial septum and vertical limb of the diagonal band as well as from the HDB entorhinal cortex, send projections to both the lateral and medial entorhinal cortex. Cholinergic neurons projecting to medial and lateral entorhinal cortex show distinct HDB topography. Whether these cholinergic projections show any layer-specifity in innervation of the parahippocampal cortical areas is not known, but physiological cholinergic responses in mouse entorhinal cortex are layer-specific (Tu et al., 2009).

New approaches such as iDISCO that allow volume imaging of the whole cholinergic network in cleared brain tissue will perhaps help to get a deeper insight in the topography of cholinergic projections in the cortex in the near future (Renier et al., 2014).

## Synaptic vs. Non-Synaptic Modulation of Neocortical Microcircuits

The classical view on cholinergic signaling in the cerebral cortex is that it is slow and aspecific, most likely volume transmission (Sarter et al., 2009; Coppola et al., 2016). Before optogenetic tools were available to selectively activate cholinergic fibers in the neocortex, indeed very few examples of fast cholinergic synaptic transmission in cortical areas existed. Interneurons in rodent hippocampus showed cholinergic fast synaptic responses mediated by nicotinic ACh receptors (Alkondon et al., 1998; Frazier et al., 1998). This sparseness of functional cholinergic synapses in the neocortex was surprising, since electron microscopy studies had revealed many examples in the cerebral cortex of rodents and primates of synaptic structures that were positive for the ACh synthesizing enzyme choline acetyltransferase (ChAT). In the cingulate cortex of the rat, 15% of cholinergic axon varicosities formed identifiable synapses (Umbriaco et al., 1994). In monkey PFC, synapses were identified on forty percent of cholinergic axon varicosities (Mrzijak et al., 1995). In human cerebral cortex, 67% of all varicosities formed identifiable synaptic specializations (Smiley et al., 1997). This may suggest that in primate, and in particular in human neocortex, direct point-to-point cholinergic synaptic transmission is more prevalent than in rodent cortex.

With optogenetic activation of BF cholinergic projections to cerebral cortex, it is now clear that ACh signaling can occur functionally through direct, point-to-point fast synapses in the cortex (Letzkus et al., 2011; Arroyo et al., 2012; Bennett et al., 2012; Kimura et al., 2014; Hay et al., 2016; Verhoog et al., 2016). Optogenetic activation of BF projections evokes barrages of inhibitory synaptic inputs to layer (L)2/3 pyramidal cells, mediated by nicotinic acetylcholine receptors (nAChRs; Arroyo et al., 2014). Very few pyramidal neurons in L2/3 express nAChRs (Poorthuis et al., 2013a) and optogenetic activation of cholinergic fibers evokes fast synaptic inputs in a small portion of L2/3 pyramidal neurons (Verhoog et al., 2016). BF fiber activation generates responses in specific cortical interneurons. L1 cells and L2/3 FS cells show mixed responses with a fast and a slow component (Letzkus et al., 2011; Arroyo et al., 2012; Verhoog et al., 2016). ACh generated depolarizing currents mediated by a mixed population of nAChRs (Arroyo et al., 2012). The slow component was blocked by dihydro-β-erythroidine (DHβE), blocker of non-α7* nAChRs. The fast component was sensitive to the α7* nAChR blocker methyllycaconitine (MLA) in both L1 and 2/3 interneurons. The inhibitory barrage on L2/3 pyramidal neurons most likely depended on the slow current component (Arroyo et al., 2012), since it was blocked by DhβE. The large trial-to trial variability of the fast component supports direct synaptic ACh transmission mediated by synaptic α7*-nAChRs. The amplitude and kinetics of the fast current was insensitive to ACh breakdown (Bennett et al., 2012; Arroyo et al., 2014). In contrast, the slow component had less trial-to-trial variability and altered upon ACh breakdown. Thus, the slow component involves diffusion of ACh over a distance, activating extra synaptic α4β2* nAChRs. The fast nAChR EPSCs result from direct transmission via synaptic or peri-synaptic α7* AChRs (Arroyo et al., 2012, 2014). Surprisingly, optogenetic activation of BF cholinergic fibers with single short light pulses triggered synaptic responses in neocortical circuits that were mediated solely by nicotinic AChRs, and not mAChRs (Letzkus et al., 2011). This contrasts with fast cholinergic control of reticular neurons in the thalamus, where activation of nicotinic and muscarinic responses result in a rapid, biphasic modulation of the membrane potential (Sun et al., 2013; Beierlein, 2017). With more sustained activation of BF projections for seconds, however, muscarinic receptors are engaged in visual cortex L2/3 interneurons, which together with nicotinic receptor activation of interneurons reduced action potential firing in pyramidal neurons that changed visual responses (Kimura et al., 2014). These experiments demonstrate that L1 and L2/3 interneurons receive both direct and diffuse cholinergic inputs in sensory cortical areas, by which the cholinergic system manipulates neocortical processing on time scales ranging from milliseconds to minutes (Arroyo et al., 2014). Thus, cholinergic control is much more deterministic, and their synaptic projections induce reliable and precise postsynaptic responses.

Direct cholinergic synaptic transmission is also found in deep layers of the neocortex. When BF inputs are activated by ChR2, prefrontal cortical L6 pyramidal neurons show an inward current that is mediated by nicotinic AChRs (Hay et al., 2016; Verhoog et al., 2016). As in L1, muscarinic receptor blockers had no effect on this current. The current was not mediated by fast α7* subunit containing nAChRs, but was completely blocked by non-α7* nACh receptor blockers (Hay et al., 2016). The slow kinetics of the current resembled that of a β2* nAChRs observed in L1 interneurons, which would suggest activation of extrasynaptic receptors. However, the onset kinetics and amplitude of these currents were not sensitive to ACh degradation. Furthermore, in low release probability conditions, response kinetics were unchanged. Finally, responsive L6 pyramidal neurons were closely apposed by cholinergic varicosities. Thus, the authors concluded that BF projections to L6 pyramidal neurons make synapses equipped with β2* nAChRs (Hay et al., 2016).

From these studies, the picture emerges that both point-to-point cholinergic synaptic transmission as well as tonic cholinergic transmission exist in the neocortex, which depends on action potential firing regimes of BF neurons. At low firing rates, only nicotinic AChRs are recruited that are predominantly located in synapses. Repetitive activity of BF cholinergic neurons recruits extrasynaptic α4β2* nAChR receptors as well as muscarinic receptors by spillover (Kimura et al., 2014; Hay et al., 2016). Thus, in the neocortex, nicotinic point-to-point synaptic transmission prevails at low firing rates of BF neurons, while a tonic extrasynaptic mode of cholinergic signaling with low temporal fidelity will occur at higher, sustained discharge frequencies of BF neurons (Kimura et al., 2014; Hay et al., 2016).

## Cholinergic Modulation of Neocortical Microcircuits Is Layer-Dependent

AChR are abundantly expressed in primate as well as rodent neocortex (Metherate, 2004; Zilles et al., 2004; Poorthuis and Mansvelder, 2013; Thiele, 2013). Both muscarinic and nicotinic AChRs alter electrical activity of target cells and can activate intracellular signaling cascades (Dajas-Bailador and Wonnacott, 2004; Gulledge and Stuart, 2005; Intskirveli and Metherate, 2012; Thiele, 2013; Yakel, 2013), despite distinct receptor mechanisms. Nicotinic AChRs form pentameric ionotropic receptors and are part of the cystine-loop superfamily of receptors which conduct sodium, potassium and calcium and depolarize membrane potentials (Gotti et al., 2006; Changeux, 2012). Muscarinic AChRs are G-protein coupled receptors that activate intracellular signaling cascades, which can lead to hyperpolarizations, depolarizations or combinations of those (Bubser et al., 2012; Dasari et al., 2017). Of the muscarinic M1 through M5 cholinergic receptors mainly M1, M2 and M4 are expressed in the neocortex (Levey et al., 1991; Bubser et al., 2012), although M4 has a considerable lower expression than the first two. In rodent neocortex, immunoreactive staining of muscarinic receptors shows strong laminar patterns (Levey et al., 1991). M1 immuno-reactivity was present in most cortical neurons and was particularly dense in L2/3 and L6. M2 protein was dense in L4 and the border of L5/6. M4 immunoreactivity was localized in L2/3, L4 and L5. In human neocortex, highest densities of M1 and M2 mAChRs were observed in superficial layers of most cortical areas (reviewed in Wevers, 2011).

Muscarinic and nicotinic AChRs also show a strong layer dependency in the cell types by which they are expressed (Gulledge et al., 2007; Poorthuis et al., 2013a). Human pyramidal cells express M1 and M2 mAChRs. M2 protein was located on apical dendrites and cell bodies of the large pyramidal cells and their dendrites in L2/3 and L5 (Wevers, 2011). L5 pyramidal cells of the rodent cortex show a biphasic response to ACh that is mediated by M1 receptors (McCormick and Prince, 1985; Xiang et al., 1998; Gulledge and Stuart, 2005; Gulledge et al., 2009; Dasari et al., 2017). In rats, cholinergic signaling through M1 mAChRs in neocortical pyramidal neurons is quite heterogeneous when comparing prefrontal, somatosensory, and visual cortex (Gulledge et al., 2007). M1 mAChRs inhibit L5 pyramidal neurons in many cortical areas via activation of an apamin-sensitive SK-type calcium-activated potassium conductance, and this was most robust in PFC L5 neurons. Pyramidal neurons in L2/3 responded less to ACh (Gulledge et al., 2007). Following the M1-mediated inhibition, rat and mouse L5 pyramidal neurons show a prolonged depolarization, which is also blocked by M1 antagonists (Dasari et al., 2017). Phasic ACh administration hyperpolarized these neurons, whereas tonic presence of ACh has opposite effects (Gulledge et al., 2007). Sustained activation of mAChRs drives prefrontal cortical pyramidal neurons and interneurons to rhythmic activity that is layer-dependent: superficial L2/3 pyramidal neurons synchronize firing at higher frequencies than deep L6 pyramidal neurons (van Aerde et al., 2009). L5 pyramidal neurons are flexible in timing their action potentials either to L2/3 or to L6 pyramidal neuron firing.

Cholinergic modulation can inhibit action potential firing of pyramidal neurons in superficial cortical layers by augmenting GABAergic inhibition through nAChRs and mAChRs (Kimura and Baughman, 1997; Disney et al., 2012; Alitto and Dan, 2013; Soma et al., 2013; Kimura et al., 2014). In rodents, application of ACh to non-fast spiking (non-FS) interneurons in layers 2/3 and 5 generates mixed responses mediated by muscarinic and nicotinic AChRs. Fast spiking (FS) interneurons in rodent neocortex generally do not show muscarinic responses (Gulledge et al., 2007), but species differences are large on this point. More than 75 percent of PV-immunoreactive visual cortical neurons in macaques, humans and guinea pigs express M1 mAChRs (Disney and Reynolds, 2014). In contrast, in rats only 25% of the visual cortex PV population is immunoreactive for M1 mAChRs. Similar to rodent cortex, non-PV interneurons in primate neocortex also express M1 mAChRs (Disney et al., 2014). M2 mAChRs are typically expressed at axons in neocortical interneurons (Disney et al., 2006).

**Figure 1:**
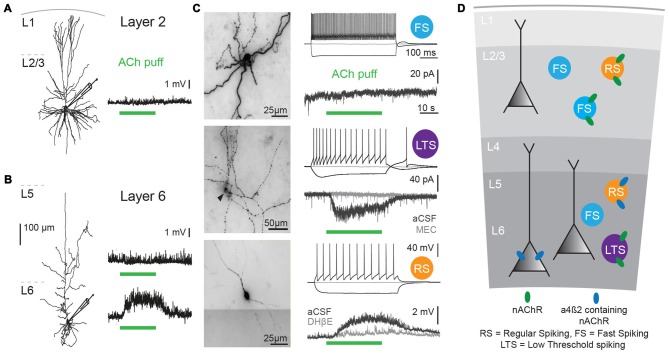
Cholinergic responses in adult human neocortex are cell type and lamina-dependent.** (A)** Left: example reconstruction of biocytin-labeled human L2/3 pyramidal neuron. Right: No change in membrane potential occurred in response to a local application (puff) of 1 mM Acetylcholine (Ach; green bar, 30 s). **(B)** Left: example reconstruction of biocytin-labeled human L6 pyramidal neuron. Right: examples of no change in membrane potential (top) and a depolarization (bottom) in response to a local application of 1 mM ACh (green bar, 30 s). **(C)** Examples of biocytin-labeled human interneurons, with action potential firing profiles in response to step current injections and voltage or current responses to local application of 1 mM ACh (green bar, 30 s). Gray traces were recorded in the presence of nicotinic acetylcholine receptor (nAChR) blockers mecamylamine (MEC, middle traces, 1 μM) or dihydro-b-erythroidine hydrobromide (DHβE, bottom traces, 1 μM). **(D)** Overview of functional nicotinic AChR expression in pyramidal neurons and the predicted expression in interneurons in different lamina of the human temporal cortex. Data figures are representing edited versions of previously published findings (Alkondon et al., 2000; Verhoog et al., 2016).

Nicotinic AChRs are highly expressed across all neocortical regions (Metherate, 2004; Millar and Gotti, 2009). Different cell types express various nAChR subunits depending on the cortical layer they are in. In addition to α4, β2 and α7 subunits, which are the most abundant neocortical nAChR subunits, the accessory α5 subunit is highly expressed in neocortex (Millar and Gotti, 2009; Counotte et al., 2012; Poorthuis et al., 2013a; Tian et al., 2014). In most cortical areas, α5 subunits are preferentially expressed in deep cortical layers (Wada et al., 1990; Tian et al., 2014), and α5 subunit expression is much lower in superficial layers (Winzer-Serhan and Leslie, 2005; Poorthuis and Mansvelder, 2013; Poorthuis et al., 2013b). Both in mouse and human neocortex, pyramidal neurons in layer 2/3 hardly ever express nAChRs: over 90% of them are devoid of nAChR currents (Figures 1A,B; Poorthuis et al., 2013a; Verhoog et al., 2016). Rodent L5 pyramidal neurons show fast nAChR currents upon ACh application, mediated by α7-containing nAChRs, whereas L6 pyramidal neurons express β2 and α5 subunit containing nAChRs that give rise to sustained inward currents that can drive action potential firing (Kassam et al., 2008; Poorthuis et al., 2013a; Verhoog et al., 2016). Human L6 pyramidal neurons also express these nAChRs and are strongly excited by them (Figures 1B,D). Interestingly, in smokers, the amplitude of nAChR currents in L6 pyramidal neurons were substantially larger than in non-smokers (Verhoog et al., 2016). Excitatory thalamocortical inputs to L5 pyramidal neurons are strongly increased by activation of presynaptic, axonal β2-containing nAChRs, as in sensory cortical areas (Lambe et al., 2003; Metherate, 2004; Kawai et al., 2007; Poorthuis et al., 2013a). Excitatory inputs to L6 pyramidal neurons are not affected by nAChR activation (Kassam et al., 2008; Poorthuis and Mansvelder, 2013). Overall activation of the prefrontal cortical network is dominated by β2-containing nAChRs and is layer specific with most prominent neuronal activation in L6, while in superficial layers, nAChRs specifically enhance inhibitory signaling (Poorthuis et al., 2013a).

Both mouse and human neocortical interneurons express functional nicotinic AChRs (Figures 1C,D; Alkondon et al., 2000; Alkondon and Albuquerque, 2004; Poorthuis et al., 2013a). In mouse PFC, fast-spiking interneurons, do not express β2 receptors. However, in contrast to fast-spiking cells in L6, parvalbumin-positive fast-spiking cells in L2/3 of the mPFC do express α7-containing nAChRs receptors (Xiang et al., 1998; Gulledge et al., 2007; Poorthuis et al., 2013a). Since PV interneurons target perisomatic compartments of pyramidal neurons, α7-containing nAChRs might regulate feedforward inhibition (Tierney et al., 2004; Rotaru et al., 2005). Somatostatin-expressing Martinotti cells in the mPFC are regulated by β2* nAChRs and hence in part might account for the strong inhibition of the pyramidal network observed during nicotinic receptor stimulation (Gulledge et al., 2007; Poorthuis et al., 2013a), which might serve to fine-tune processing of synaptic inputs arriving at distal dendrites of pyramidal neurons.

In sensory cortical areas, such as auditory and visual cortex, VIP-positive neurons in superficial layers are recruited by cholinergic inputs that activate nicotinic AChRs (Porter et al., 1999; Letzkus et al., 2011; Bennett et al., 2012; Poorthuis et al., 2014). Non fast-spiking (NFS) interneurons form a heterogeneous group of interneurons and half of them express β2-containing nAChRs, sometimes accompanied by α7-containing nAChRs (Poorthuis et al., 2013a). β2-containing nAChR expression of this cell type was found across all cortical layers, indicating that they perform similar roles across these microcircuits to fine-tune pyramidal function. Since VIP-positive interneurons preferentially inhibit somatostatin-positive and PV-positive interneurons, nAChRs can augment inhibitory as well as disinhibitory signals to neocortical pyramidal neurons. The phenomena of augmented inhibition and increased disinhibition by nicotinic AChR activation has been observed both in mouse as well as in human neocortex (Alkondon et al., 2000).

Cholinergic receptors are thus placed in excellent positions to rapidly modulate various inhibitory circuit motifs (Tremblay et al., 2016): feed-forward inhibition, lateral inhibition and disinhibition. Thereby, the distributed laminar expression pattern of nicotinic and muscarinic receptors can facilitate specific and instantaneous changes in the direction of information flow within cortical circuits (Xiang et al., 1998; Gulledge et al., 2007; Poorthuis et al., 2013a). This will depend on the cortical layer in which they are located and the temporal pattern by which these receptors are activated, but can occur on time scales of milliseconds to minutes.

## Layer-Dependent Modulation of Cortical Synaptic Plasticity

Cholinergic modulation of cerebral cortical circuits is not limited to transient changes in cellular and synaptic activity. Both short-term and long-term synaptic plasticity can be altered by cholinergic receptor activation by BF inputs. The cellular and sub-cellular location of cholinergic receptors not only affects neuronal circuitry excitability, but it will also determine how glutamatergic synapse plasticity is affected by cholinergic inputs (Verhoog et al., 2016). For instance, nAChRs containing α5 subunits expressed in L6 pyramidal neurons regulate short-term plasticity at L6 glutamatergic synapses (Hay et al., 2016). Nicotinic AChRs on presynaptic terminals transiently augment synaptic glutamate release (McGehee et al., 1995; Gray et al., 1996), and presynaptic α7 subunit-containing nAChRs with high calcium permeability, induce long-term potentiation of glutamatergic synapses in several brain areas (Mansvelder and McGehee, 2000; Gu and Yakel, 2011).

Modulation of synaptic plasticity by muscarinic receptors has been found in hippocampal circuits. Low concentrations of mAChR agonists can modulate plasticity of glutamatergic synapses onto hippocampal pyramidal neurons (Shinoe et al., 2005), by inhibiting potassium channels (Buchanan et al., 2010). M1 mAChRs directly excite CA1 pyramidal neurons and induce a robust strengthening of glutamatergic synapses in CA1 pyramidal neurons (Dennis et al., 2016). Whether these muscarinic mechanisms also modulate synaptic plasticity in the neocortex remains to be established. Spike-timing-dependent plasticity is altered by recruitment of mAChRs and presynaptic nAChRs (Ji et al., 2001; Ge and Dani, 2005; Gu and Yakel, 2011). Activity of nAChRs bi-directionally alters plasticity, and the sign of synaptic change depended on timing and localization of nAChR activation. Dendritic α7 nAChRs boost short-term plasticity into long-term plasticity. When nAChRs on neighboring interneurons were activated, plasticity was blocked (Ji et al., 2001). In L6 of the entorhinal cortex, non-α7 nAChRs can also boosted short-term to long-term potentiation (Tu et al., 2009), but mechanisms and locations of nAChR remain to be elucidated.

**Figure 2:**
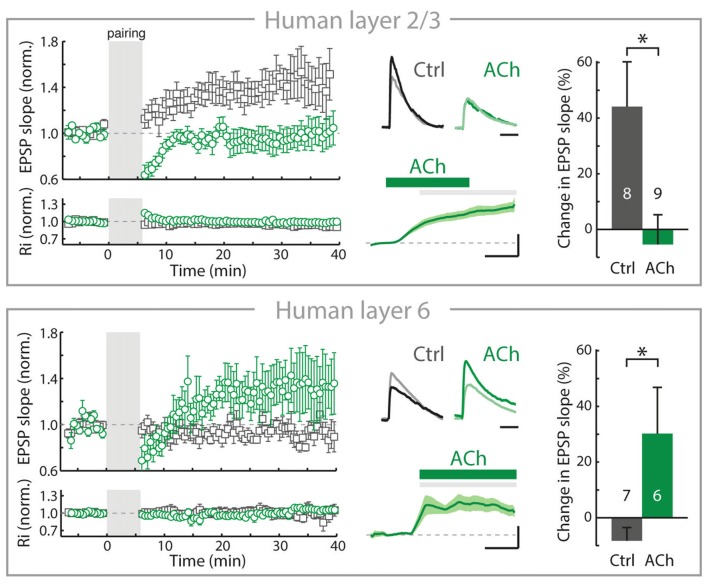
In adult human neocortex, ACh alters long-term synaptic plasticity of glutamatergic synapses in opposite directions in superficial and deep layers. Top, left: In adult human neocortical L2/3 pyramidal neurons in control conditions (open gray square) and experiments where ACh was present in the bath during long-term potentiation induction (pre- and postsynaptic activity pairing, open green circle). Top, middle: top traces: example EPSP waveforms recorded during baseline (light color) and 20–25 min. after pairing (dark color), for tLTP experiments with and without ACh present in bath during pairing. Horizontal scale bar: 30 ms; vertical scaling as below. Bottom traces: membrane potential change over course of pairing period (gray shading) relative to baseline for experiments where ACh was washed-in during pairing. Scale bars, 3 mV, 2 min. Top, right: summary bar chart showing change in EPSP slope of control LTP and ACh LTP experiments in human L2/3 neurons. Bottom, as in top figure, for nAChR-bearing human L6 pyramidal neurons. Note that in contrast to L2/3, in L6 long-term potentiation of human glutamatergic synpases is increased by ACh (*p ≤ 0.05). Figure is modified from Verhoog et al. (2016), no permission required.

Over the past decade, we have found that in the rodent PFC, the rules of inducing plasticity of glutamatergic synapses are altered by cholinergic inputs from the BF depending on the cortical layers, resulting from a layer-and cell-type specific expression of nAChRs (Couey et al., 2007; Goriounova and Mansvelder, 2012; Verhoog et al., 2016). Recently, we identified similar mechanisms in human neocortex: Cortical lamina-specific distributions of nAChRs induce opposite manipulation of synaptic plasticity by ACh in superficial and deep layers (Figure 2; Verhoog et al., 2016).

In the rodent PFC, despite the robust nAChR modulation of thalamic excitatory inputs (Lambe et al., 2003; Couey et al., 2007; Poorthuis et al., 2013a), inhibitory synaptic transmission is increased and the excitability of L2/3 and L5 pyramidal neuron is reduced by nAChRs located on GABAergic interneurons. Activation of these nAChRs on interneurons by BF inputs suppresses long-term potentiation of glutamatergic synapses by reducing calcium signaling in pyramidal neuron dendrites of L2/3 and L5 (Couey et al., 2007; Goriounova and Mansvelder, 2012; Verhoog et al., 2016). These mechanisms are also in place in the rodent insular cortex (Sato et al., 2017). In contrast to superficial layers, nAChRs do not modulate inhibitory GABAergic and excitatory glutamatergic transmission in L6 pyramidal neurons. Instead, L6 pyramidal neurons are directly activated by postsynaptic nAChRs (Kassam et al., 2008; Bailey et al., 2012; Poorthuis et al., 2013a). Activation of these postsynaptic nAChRs on L6 pyramidal neurons by ACh release from BF inputs increases glutamatergic synaptic plasticity by augmenting calcium influx in pyramidal neuron dendrites (Verhoog et al., 2016). Thus, in L6 glutamatergic synaptic plasticity is modulated in the opposite direction from superficial layers by endogenous ACh, which oppositely modulates dendritic calcium signals in superficial and deep layer pyramidal neurons. These mechanisms also exist in human brain: cortical lamina-specific nAChR expression drives opposite cholinergic modulation of synaptic plasticity in superficial and deep layers (Figure 2). Thus, the functional organization of cortical microcircuit modulation by the cholinergic system is most likely similar in rodent and human neocortex (Verhoog et al., 2016).

In the rodent PFC, fast synaptic cholinergic synaptic inputs mediated by nAChR containing β2 and α5 subunits are received by L6 pyramidal neurons (Hay et al., 2016), and these inputs can enhance synaptic plasticity (Verhoog et al., 2016). Since distinct BF nuclei preferentially target superficial or deep layers (Bloem et al., 2014), distinct BF cholinergic neuron populations may innervate superficial interneurons and L6 pyramidal neurons. The combination of the cell-type and lamina-specific distribution of cortical nAChRs taking part in ACh synaptic transmission (Poorthuis et al., 2013a; Arroyo et al., 2014; Hay et al., 2016), enables a fine spatial and temporal control of cortical signaling and plasticity by BF cholinergic neurons.

## Conclusion and Future Directions

The evidence thus presents us with a landscape of cholinergic modulation of the cerebral cortex that is much more refined than previously considered, both on a spatial as well as a temporal scale. From various retrograde and anterograde anatomical methods it is now becoming clear that the BF cholinergic system specifically targets sub-regions of brain areas, as found in both prefrontal cortical subareas as well as rhinal cortical subareas (Bloem et al., 2014; Kondo and Zaborszky, 2016). Based on topographic location of cholinergic neurons, not only specific parts of brain areas are targeted, but superficial and deep cortical layers are distinctly innervated as well. Both neocortical pyramidal and interneurons can receive fast point-to-point cholinergic synapses that use nicotinic AChRs. Extrasynaptic nicotinic and muscarinic AChRs are predominantly recruited during repeated firing by BF cholinergic neurons. Thereby, both specific rapid changes in microcircuit activity can be induced on a millisecond time scale, as well as cortical state changes that last minutes or more. The striking observation that in human neocortex, 67% of cholinergic varicosities form synaptic specializations (Smiley et al., 1997), in contrast to only 15% in rodent neocortex (Umbriaco et al., 1994), may suggest that direct point-to-point cholinergic synaptic transmission is highly prevalent in human cortex, but this awaits further testing. Both in rodent and human neocortical microcircuits, the cell-type specific distribution of nicotinic and muscarinic support a layer-specific modulation of circuit motifs and synaptic plasticity. Although the human neocortex shows structural similarities to the rodent neocortex, many striking differences in cellular and synaptic structure and function have been uncovered in recent years (presented in detail in Table 1, Molnár et al., 2008; Verhoog et al., 2013; Testa-Silva et al., 2014; Mohan et al., 2015; Eyal et al., 2016). The fact that a layer-specific pattern of nAChR expression underlies distinct cholinergic modulation of glutamatergic synaptic plasticity in human neocortex as well (Verhoog et al., 2016), raises the possibility that in the human brain too, cholinergic modulation of cortical information processing may operate on a highly detailed scale, both in cortical space and time. Novel approaches will be needed to be able to test whether functional fast cholinergic transmission is prevalent in human neocortex, as is suggested by the ultrastructural analysis.

**Table 1:** Cross-species comparison of the neocortical cholinergic system.

Anatomy of cholinergic projections	Rodent	Monkey	Human
Topographical organization of cholinergic projections from the BF to the cortex	Yes (1,2,3)	-	Yes (4)
Dominant BF origin of neocortical cholinergic innervation	Nucleus Basalis, SI (5,6,7)	Nucleus Basalis, SI (Ch4) (8)	Nucleus Basalis, SI (Ch4) (8)
**Cholinergic synapses**			
Percentage of varicosities on cholinergic axons that from identified synaptic structures in EM	15% (Cingulate cortex) (9)	40% (PFC) (10)	67% (Temporal cortex) (11)
**Muscarinic acetylcholine receptors**			
Layer dependent expression of muscarinic receptors M1–M5	Yes (12,13)	Yes (14)	Yes (14)
Expression of M1 mAChR	L2/3 and 6 (14)	L1–6 (15)	Superficial layers (14)
Expression of M2 mAChR	L4 and border L5/6 (14)	L1–6 (23)	Superficial layers (14)
Expression of M1 mAChR in pyramidal neurons in the neocortex	L5 (12)	-	-
Expression of M1 mAChR in non-PV interneurons in the neocortex	Yes (14)	Yes (15)	-
Percentage of PV-interneurons in the visual cortex that express M1 mAChR	25% (15)	75% (15)	-
Expression of M2 mAChR in non-PV interneurons in the neocortex	No (12)	Yes (23)	-
Percentage of PV-interneurons in the visual cortex that express M2 mAChR	-	20%–70% (23)	-
**Nicotinic acetylcholine receptors**			
Expression of active nAChRs in pyramidal and interneurons in the neocortex	Yes (16,17,18,19,20)	-	Yes (20,21,22)
Layer specific expression of nAChRs in pyramidal neurons in the neocortex	Yes (20)	-	Yes (20)
Increased dishinhibition by activation of nAChR in the neocortex	Yes (22)	-	Yes (21)
**Plasticity**			
Layer specific modulation of plasticity in the human neocortex by nAChRs	Yes (20)	-	Yes (20)

References: 1,2,3 (Bloem et al., 2014; Woolf and Butcher, 2011; Zaborszky et al., 2015), 4 (Mesulam, 2013), 5,6,7 (Lamour et al., 1982; Price and Stern, 1983; Rye et al., 1984), 8 (Mesulam and Geula, 1988), 9 (Umbriaco et al., 1994), 10 (Mrzijak et al., 1995), 11 (Smiley et al., 1997), 12,13 (Gulledge et al., 2007; Levey et al., 1991), 14 (Wevers, 2011), 15 (Disney et al., 2014), 16,17,18,19,20 (Counotte et al., 2012; Millar and Gotti, 2009; Poorthuis et al., 2013a; Tian et al., 2014; Verhoog et al., 2016), 21,22 (Alkondon et al., 2000; Alkondon and Albuquerque, 2004), 22 (Letzkus et al., 2011), 23 (Disney et al., 2006).

The findings that ACh can act both on a slow time scale and in fast synaptic transmission, in both a volume transmission mode and point to point cholinergic synapse level extends our views on how ACh can modulate microcircuit function in the cortex. New *in vivo* optogenetic approaches should test what the role of these different signaling modes is in behavior. Activating or inhibiting cholinergic projections on a fast time scale should bring us in a position to unravel how this correlates with attention and memory performance. Disentangling the contributions of fast and slow signaling modes could also provide a novel view on disorders in which cholinergic signaling is affected, such as Alzheimer disease. The binding of Aβ to α7 nAchRs (Nagele et al., 2002; Lamb et al., 2005; Gu and Yakel, 2011) may differentially affect synaptic vs. slower cholinergic signaling modes.

## Author Contributions

HDM conceived the story line of the review. JO, MVB, AL and HDM contributed to the writing of the manuscript. JO made Figure 1, MBV made Figure 2. All authors commented on the text and figures.

## Conflict of Interest Statement

The authors declare that the research was conducted in the absence of any commercial or financial relationships that could be construed as a potential conflict of interest.
